# Efficacy and safety of acupuncture combined with western medicine for anxiety: A systematic review protocol

**DOI:** 10.1097/MD.0000000000021445

**Published:** 2020-07-31

**Authors:** Aihua Tan, Miyuan Wang, Jia Liu, Kailin Huang, Disha Dai, Lei Li, Heyuan Shi, Ping Wang

**Affiliations:** a Hubei University of Chinese Medicine, Wuhan, Hubei; b Beijing University of Chinese Medicine, Beijing; c People's Hospital of Wuhan University, Wuhan, Hubei, China.

**Keywords:** acupuncture, anxiety, protocol, systematic review, western medicine

## Abstract

**Background:**

As a common clinical mental disorder, the prevalence rate of anxiety disorder increased yearly, devastating both physical health and social-economic prospect. The most common treatment relied on the use of western medications which is yet to fulfill ideal performance. While acupuncture is adopted as a treatment for anxiety disorders, the combination treatment of acupuncture and western medicines becomes more acknowledged. Albeit a spike in related literatures, the curative effect and safety of the treatment are still in lack of evidence. Therefore, this systematic review and meta-analysis protocol is planned to evaluate the efficacy and safety of the combination treatment of acupuncture and western medications.

**Methods:**

Six English databases (PubMed, Web of science, Medline, EBASE, Springer Cochrane Library and WHO International Clinical Trials Registry Platform) and four Chinese databases (Wan fang Database, Chinese Scientific Journal Database, China National Knowledge Infrastructure Database (CNKI) and Chinese Biomedical Literature Database) will be searched normatively according to the rule of each database from the inception to June 1, 2020. Two reviewers will independently conduct article selection, data collection, and risk of bias evaluation. Any disagreement will be resolved by discussion with the third reviewer. Either the fixed-effects or random-effects model will be used for data synthesis based on the heterogeneity test. The change in the scores on the Hamilton Anxiety Scale (HANA) and the self-rating anxiety scale (SAS) will be used as the main outcome measure, quality of life scale (SF-36), changes of symptoms in TCM, hormone levels and clinical global impression (CGI) as the secondary outcome. treatment emergent symptom scale (TESS), general physical examination(temperature, pulse, respiration, blood pressure), Routine examination of blood, urine and stool, Electrocardiogram, Liver and kidney function examination as the security indexes. RevMan 5.3.5 will be used for meta-analysis.

**Results:**

This study will provide high-quality evidence to assess the effectiveness and safety of acupuncture combined with western medicine for anxiety.

**Conclusion:**

This systematic review will explore whether acupuncture combined with western medicine is an effective and safe intervention for anxiety.

**Ethics and dissemination:**

Ethical approval is not required for this study. The systematic review will be published in a peer-reviewed journal, presented at conferences, and will be shared on social media platforms. This review will be disseminated in a peer-reviewed journal or conference presentation.

**PROSPERO registration number:**

PROSPERO CRD42020149746.

## 1 Introduction

Anxiety disorders are one of the most common psychological disorders, characterized by excessive fear, anxiety, or avoidance of an array of external and internal stimuli.^[1,2]^ In a systematic review of 87 prevalence studies across 44 countries, an estimated 7.3% (1 in 14 people) of the worldwide population is suffering from anxiety at any given time.^[3]^ Base on the World Mental Health Survey, the lifetime prevalence of anxiety disorder is estimated to range from 4.8% in China to 31% in the United States.^[4]^ Often begin during childhood,^[5]^ anxiety disorder prompted severe disabling effects on patient's social, personal, and occupational functioning, as well as leading to a significant loss in quality of life and with enormous social cost.^[1,6]^ According to the Global Burden of Disease study, anxiety disorders are the sixth leading cause of disability in both high-income and low-income countries, with the highest burden rate between age 15 to 34.^[7]^

Pharmacotherapy (western medicine therapy) has always been considered the primary treatment for anxiety disorders.^[8]^ However, the following concerns hinder it from being ideal for treating anxiety disorders: First of all, limited efficacy. Previous studies have shown that at least one third of the patients with anxiety disorders have poor drug responses.^[9]^ Second, various adverse effects derived from medications. First-line anti-anxiety drugs such as selective serotonin-reuptake inhibitors (SSRIs) and serotonin–noradrenaline-reuptake inhibitors (SNRIs) are proved to initiate adverse reactions such as: nausea, diarrhea, dizziness, headache, insomnia, emotional symptoms and sexual dysfunction, etc.^[10–13]^ While second-line medications such as benzodiazepines drugs have a greater drug dependence potential with both dosages and applications restricted, altogether lowering patients’ compliances.^[14]^ Third, a relatively slow outcome. In most cases, persistent symptoms of anxiety disorders on the majority of the patients remained after evaluating 6 to 12 weeks of treatment, despite a thorough pharmacotherapy.^[15]^ Last of all, a high recurrence rate. A 2-year follow-up study on depression and anxiety disorder conducted in the Netherlands has disclosed a 35% recurrence rate on anxiety disorder all the while antidepressants were continuously taken.^[16]^ Therefore, proposals on more effective, and safer approaches for patients with anxiety disorder are under plausible urgency.

Acupuncture treatment is an ancient Chinese therapy that has guarded the Chinese healthcare system for over 3000 years while gaining its reputation worldwide.^[17–19]^ The theory of acupuncture mainly derived from the concept of holism, Zang-Fu viscera and meridians and collaterals in traditional Chinese medicine, and the therapeutic effects are achieved by regulating the body's Qi, blood, Yin and Yang to equilibrium.^[20]^ Acupuncture gained popularity in the treatment of anxiety disorders during recent years.^[21]^ Studies have proved its ability to change prefrontal cortex activity, regulates plasma corticosteroid, adrenocorticotrophic hormone, and platelet 5-HT levels, etc. to relieve anxiety.^[22,23]^ The combination therapy of acupuncture and western medicines is believed to enhance the therapeutic effect, speeds onset time, minimizes adverse reactions from western medicines, and reduce the recurrence rate of anxiety disorder.^[24–28]^ A decent amount of systematic reviews and meta-analysis had revealed the efficacy and safety of acupuncture for the treatment of anxiety disorders.^[29–31]^ However, all research regarded only acupuncture treatment on patients albeit western medicines remained the standard treatment of anxiety disorders. The lack of a meta-analysis focusing on acupuncture combined with western medicines in the treatment of anxiety disorder had prompted this comprehensive systematic review and meta-analysis. The evidence of RCTs regarding acupuncture in combination with western medicines on the treatment of anxiety disorders will be evaluated.

## 2 Methods

### 2.1 Study registration

This review protocol is registered in the PROSPERO International Prospective Register of systematic reviews, registration number ***CRD42020149746***. Available from: http://www.crd.york.ac.uk/PROSPERO/display_record.php?ID=CRD42020149746, and has been reported following the preferred reporting items for systematic reviews and meta-analyses guidelines.^[32]^

### 2.2 Inclusion criteria for study selection

#### 2.2.1 Types of studies

All relevant randomized controlled trials (RCTs) in English and Chinese will be included. While Non-RCTs, quasi-RCTs, cohort studies, reviews, case reports, experimental studies, expert experience, the data of the included study is missing or incomplete, and duplicate publications will be excluded to ensure the quality of this systematic review.

#### 2.2.2 Types of participants

Participants of different age groups with anxiety disorders could be included in the study, regardless of nationality, race, gender, occupation, and educational background. While the cause of anxiety is not limited, experimental objects which included patients with depression and schizophrenia would be excluded.

#### 2.2.3 Types of interventions

This study focuses on the clinical trial (RCTs) of anxiety disorder treated under a combination treatment of acupuncture and western medicines. The results are anticipated to aid clinicians. All trials with an assessment of the combination treatment mentioned above will be included, while studies of control group could only use western medicines as the sole treatment.

#### 2.2.4 Types of outcome measures

##### 2.2.4.1 Primary outcomes

The primary outcomes are the Hamilton anxiety scale (HAMA) and the self-rating anxiety scale (SAS).

##### 2.2.4.2 Secondary outcomes

The secondary outcomes of this review mainly include the following aspects:

1. Quality of life scale (SF-36)
2. Changes of symptoms in TCM, and Hormone levels.
3. Clinical global impression (CGI).

##### 2.2.4.3 Security Index

1. Treatment emergent symptom scale (TESS).
2. General physical examination (temperature, pulse, respiration, blood pressure).
3. Routine examination of blood, urine and stool.
4. Electrocardiogram.
5. Liver and kidney function examination.

### 2.3 Data sources

Six English databases (PubMed, Web of science, Medline, EBASE, Springer Cochrane Library and WHO International Clinical Trials Registry Platform) and four Chinese databases (Wanfang Database, Chinese Scientific Journal Database, China National Knowledge Infrastructure Database and Chinese Biomedical Literature Database) will be searched in accordance to the rule of the respective database from the inception to 1st June, 2020.

### 2.4 Searching strategy

Search strategy will be built in accordance to the guidelines from the Cochrane handbook. The Search strategy for PubMed is shown in Table 1, which included all search terms, and similar strategies will be built and applied for other electronic databases.

**Table 1:**
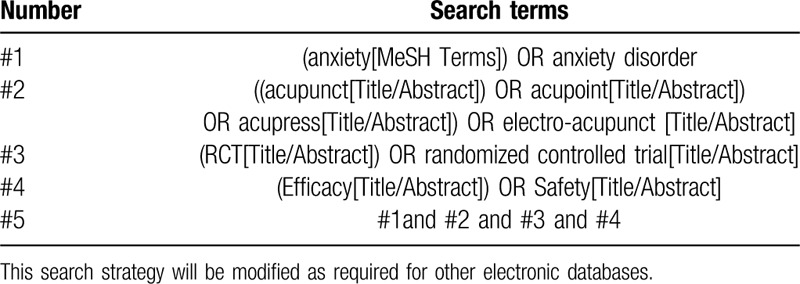
The search strategy for PubMed.

### 2.5 Data collection and analysis

#### 2.5.1 Selection of studies

**Figure 1:**
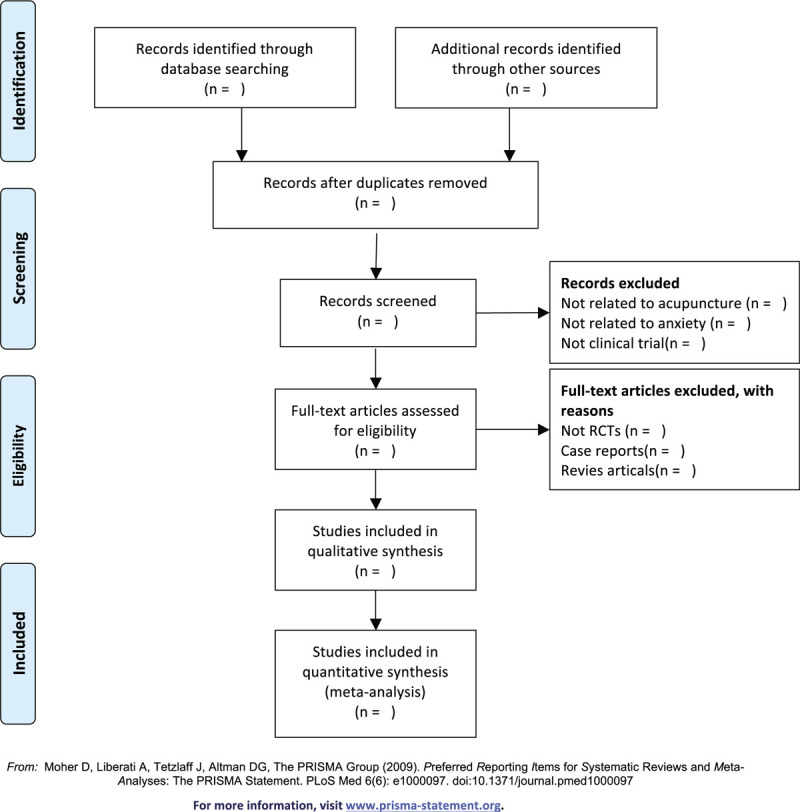
The PRISMA flow chart of the selection process. PRISMA = systematic reviews and meta-analysis protocol.

The basic process of including literature will be pursued in reference to the Cochrane Collaboration System Evaluator's Manual (5.1.0). Relevant literatures will be obtained from specified databases, later imported into a database created by Endnote X7. Duplicate documents will be screened out through this process. Independent screening of titles, abstracts, and keywords of all retrieved records will be performed by two researchers. The name of the study, author, publishing year, country, database, and justification of the study meeting eligibility criteria to be therefore included in the review will be documented within an Excel spreadsheet. Reasons of inclusion and exclusion (PICOS) are disclosed in a spreadsheet during abstract screening and full-text evaluation. A third researcher will be required on making the final decision to resolve any disagreement among the two researchers on literatures. The screening flow diagrams of this study will be shown in Figure 1.

#### 2.5.2 Data extraction and management

Two independent reviewers will extract the data of interest from the eligible study and fill in the data collection sheet. If consensus on data extraction failed to reach by discussion, the decision will be made by the third reviewer. Microsoft Excel 2013 will be used for data and information management. We will extract the following data:

1. The basic characteristics of RCT: title, 1st author, publishing year, country, and the journal.
2. Participants’ characteristics: average age, gender, sample size, inclusion and exclusion criteria, baseline situation, type, and criteria for the classification of anxiety.
3. Interventions: treatment duration, study design, randomization, allocation concealment, and blinding methods
4. Comparators: western medicines
5. Outcomes: measures, primary and secondary outcomes, security indexes, and follow up.

#### 2.5.3 Assessment of risk of bias

Cochrane bias risk tool (RevMan5.3.5) will be used to evaluate the risk of bias, and the following 6 domains will be assessed: random sequence generation, allocation concealment, blinding, incomplete outcome data, selective reporting, and other bias. Each potential trial of bias will be graded as high, low, and unclear. When the 2 independent reviewers failed to reach a consensus on the risk of bias assessment by negotiation, a third reviewer will make a final decision.

#### 2.5.4 Measures of treatment effect

Mean differences (MD) or standard mean difference (SMD) with 95% CIs will be used as continuous data, and the dichotomous outcomes will be estimated by the risk ratio (RR) with 95% confidence intervals (CIs).

#### 2.5.5 Unit of analysis issues

Only the 1st experimental period data of crossover trials will be extracted in order to minimize carryover effects. For trials regarding multiple interventions, all relevant experimental groups and control groups within the trial will be combined into a single group to avoid unit-of-analysis error.

#### 2.5.6 Management of missing data

For missing data, we will first try to contact the original author. If the data failed to be provided on request, it will be excluded from the study.

#### 2.5.7 Assessment of heterogeneity

Heterogeneity will be assessed by visual inspection of the forest plots and detected by standard χ^2^ test and I^2^ test. When *P* > .1, I^2^ < 50%, it will be considered as no significant heterogeneity between the trials, and the fixed effect model will be applied for statistics, otherwise, the random effect model will be chosen. When heterogeneity occurs, sensitivity analysis or Meta regression will be performed to assess the source of heterogeneity.

#### 2.5.8 Assessment of reporting biases

If ten or more studies are included in the meta-analysis, funnel plots and Egger test will be used to evaluate the reporting bias. The trim and fill method will be applied to identify and correct asymmetric funnel arising from publication bias, if appropriate.^[33]^

#### 2.5.9 Data synthesis

Data analysis and synthesis will be performed using RevMan version 5.3 software provided by the Cochrane Collaboration. The software will be used to obtain forest plots and test the heterogeneity between the included studies. Risk ratio (RR) with 95% CIs will be used for dichotomous data, while the continuous data will be analyzed by mean difference (MD) or standard MD (SMD) with 95% CIs.

#### 2.5.10 Subgroup analysis

When heterogeneity is detected, subgroup analysis will be used (e.g., different types of western medicines therapies, patient conditions, research quality, publication age, and participation population) to spot the source of heterogeneity.

#### 2.5.11 Sensitivity analysis

In trials with sufficient data, sensitivity analyses will be taken to test the robustness and reliability of the results. Our sensitivity analysis will be based on heterogeneity. We may perform sensitivity analysis by excluding certain low-quality studies or unblinded studies when heterogeneity occurs.

## 3 Discussion

Anxiety disorders are one of the most common mental health concerns that frequently begin in childhood and can have severely disabling effects on social, occupational, and other areas of functioning. As the most important treatment for anxiety disorders, western medicine has the defects of slow onset, limited efficacy, severe adverse effects, and high recurrence rate.^[9–15]^ As a complementary and alternative treatment, acupuncture in combination with western medicines is able to accelerate the onset time, improves therapeutic effect, minimizes adverse effects, and reduces the recurrence rate.^[24–28]^ There is, however, insufficient evidence to prove any significant efficacy of the combination treatment of acupuncture and western medicine on anxiety disorders. This study aimed to review all randomized controlled trials comparing the combination treatment of acupuncture with western medicine against sole western medicine treatment on anxiety disorders systematically. This systematic review and meta-analysis will provide a convincing conclusion to justify the efficacy and safety of the combination treatment of acupuncture and western medicine on anxiety disorder. The conclusion of this review is anticipated to assist clinicians regarding the treatments of anxiety disorder and benefits corresponding patients. These clues and conclusion are hoped to encourage researchers to conduct further research on the subject.

## Author contributions

AHT, MYW, JL, HYS, and PW conceived and designed the protocol, and AHT drafted the protocol manuscript. AHT developed the search strategy, with input from KLH and DSD. MYW, JL and LL planned the data extraction. AHT, JL and MYW planned the quality appraisal of all included studies. AHT, MYW, JL, KLH, DDS, LL, HYS, and PW, critically revised the manuscript for methodological and intellectual content. All authors approved the final version.

**Conceptualization:** Aihua Tan, Miyuan Wang, Jia Liu, Heyuan Shi, Ping Wang.

**Data curation:** Miyuan Wang, Jia Liu, Lei Li, Kailin Huang.

**Formal analysis:** Miyuan Wang, Disha Dai.

**Project administration:** Aihua Tan, Miyuan Wang, Jia Liu.

**Supervision:** Aihua Tan, Heyuan Shi, Miyuan Wang, Jia Liu.

**Writing – original draft:** Aihua Tan, Miyuan Wang, Jia Liu.

**Writing – review & editing:** Aihua Tan, Ping Wang.
